# Nanoparticles as Drug Delivery Systems in Cancer Medicine: Emphasis on RNAi-Containing Nanoliposomes

**DOI:** 10.3390/ph6111361

**Published:** 2013-11-04

**Authors:** Mónica Rivera Díaz, Pablo E. Vivas-Mejia

**Affiliations:** 1 Department of Biochemistry, University of Puerto Rico, San Juan, Puerto Rico 00935, USA; E-Mail: monica.rivera3@upr.edu; 2 Comprehensive Cancer Center & Department of Biochemistry, University of Puerto Rico, San Juan, Puerto Rico 00935, USA

**Keywords:** nanoparticles, nanoliposomes, RNAi, miRNA, siRNA

## Abstract

Nanomedicine is a growing research field dealing with the creation and manipulation of materials at a nanometer scale for the better treatment, diagnosis and imaging of diseases. In cancer medicine, the use of nanoparticles as drug delivery systems has advanced the bioavailability, *in vivo* stability, intestinal absorption, solubility, sustained and targeted delivery, and therapeutic effectiveness of several anticancer agents. The expansion of novel nanoparticles for drug delivery is an exciting and challenging research filed, in particular for the delivery of emerging cancer therapies, including small interference RNA (siRNA) and microRNA (miRNAs)-based molecules. In this review, we focus on the currently available drug delivery systems for anticancer agents. In addition, we will discuss the promising use of nanoparticles for novel cancer treatment strategies.

## 1. Introduction

The engineering, characterization, synthesis, and use of materials and devices of 100 nanometers or less is called nanotechnology [1]. The application of nanotechnology to medicine, designated as nanomedicine has greatly accelerated the diagnosis, imaging and treatment of many diseases. In cancer medicine, nanotechnology has become a potential application for the development of nanoparticles as drug delivery systems. Classical very potent chemotherapeutic agents, including camptothecin, taxenes, platinating agents, doxorobucin, and nucleoside and nucleotide analogs have been used against several tumor types for several decades. However, they have the disadvantage of affecting both tumor cells and normal cells, with the concomitant secondary effects including, cardiotoxicity, cytotoxicity, neurotoxicity, nephrotoxicity, and ototoxicity [1,2,3]. Some of these chemotherapeutic-associated problems have been solved by the use of nanoparticle formulations of these drugs. The most important advantage of these novel formulations is that they preferentially target tumor cells by the enhanced permeability and retention (EPR) phenomenon exhibited by solid tumors compared with normal tissues [4]. In addition, nanoparticles as therapeutics carriers have other unique properties of higher therapeutic efficacy, lower toxicity and the ability to encapsulate and deliver poorly soluble drugs [1].

The elaboration of nanoparticles of uniform shape, size and composition is a dynamic growing research field in cancer medicine. Novel improved biodegradable and biocompatible nanoparticle formulation with increasing bioavailability, *in vivo* stability, intestinal absorption, solubility, sustained and targeted delivery to site of action combined with therapeutic effectiveness, are being developed [5].

In this review, we discuss the characteristics of various nanotechnology-based drug delivery systems including, carbon nanotubes, dendrimers, micelles, quantum dots, fullerenes, nanofibers, metal-based nanoparticles, and nanoliposomes. We will emphasize on the application of nanoliposomes for the delivery of small interference RNA (siRNA) and microRNA (miRNAs)-based molecules including their use in current clinical trials for cancer treatment.

## 2. Common Nanoparticles Used in Cancer Medicine

Nanotechnology has opened a window for the development of diverse organic and inorganic drug carriers, known as nanoparticles. Source materials include phospholipids, lactic acid, chitosan, dextran, polyethylene glycol (PEG), cholesterol, carbon, silica, and some metals [1,6,7,8,9,10]. The surface of nanoparticles is further modified by covalent conjugation with small functional groups that increase their targeting potential. Functional groups that improve the nanoparticle specificity include folate, antibodies, aptamers, and the tripeptide Arg-Gly-Asp (RGD) [11]. In this section, we will discuss the characteristics of the major nanoparticle platforms used as drug delivery systems.

### 2.1. Polymeric Nanoparticles

Polymeric nanoparticles are colloidal solid particles prepared from biodegradable polymers such as chitosan and collagen or non-biodegradable polymers such as poly(lactic acid) (PLA) and poly(lactic co-glycolic acid) (PLGA) [9,12,13,14,15]. Their small size (50–300 nm) allows these particles to penetrate capillaries and to be taken up by the cells, increasing the accumulation of the drug at the target site of action [9]. The majority of these compounds are formulated through a spontaneous self-assembly process using block polymers of two or more polymeric chains with different hydrophilicity [1]. They are considered promising nanocarriers for drug delivery because they can improve the specificity to the target site of action by changing their physicochemical properties and pharmacokinetics [12,16]. The stability of PLGA nanoparticles can be further improved by coating them with PEG [7]. For example, Danhier *et al.* used paclitaxel-loaded PEG-PLGA-based nanoparticles grafted with RGD peptide, and found that the target nanoparticles reduced tumor growth more efficiently, and prolonged survival times of mice, compared with non-targeted nanoparticles [8]. A different very promising polymeric nanoparticle is the chitosan based-nanoparticles [17,18]. Chitosan is a natural polymer obtained by the partial N-deacetylation of chitin, the second most abundant polysaccharide in Nature [17,18]. Doxorubicin (DOX)-loaded chitosan nanoparticles, and DOX-loaded anti-human growth factor receptor 2 (Her2)-surface modified chitosan nanoparticles have been proposed [19,20]. A modified PLGA nanoparticle containing chitosan through physical adsorption and chemical binding methods has also been described [21]. However, more *in vivo* studies are needed to demonstrate the efficacy and safety of PLGA and chitosan nanoparticles as drug carriers.

### 2.2. Polymeric Micelles

Polymeric micelles are made by amphiphilic block copolymers such as poly (ethylene oxide)-poly(β-benzyl-L-aspartate) and poly (*N*-isopropylacrylamide)-polystyrene. Micelles of less than 100 nm assembled with a hydrophobic core and hydrophilic shell are commonly used as drug carriers [9,12,22]. The small size of micelles, allows the specific accumulation in the pathologic tissue [12]. Their hydrophobic core and hydrophilic shell make micelles potent nanocarriers for poorly water soluble anticancer drugs, including paclitaxel and docetaxel [23,24]. One particular feature of micelles is that the amount of drug released can be controlled by an external stimulus like pH, temperature, ultrasound or certain enzymes [25]. Other unique properties of polymeric micelles are that they are easily modified with small functional groups that increase their targeting potential. For example, Kagaya *et al.* evaluated gene delivery efficacy to vascular lesions using cyclic RGD (cRGD)-PEG-polyplex micelles [26]. They found that cRGD-PEG-polyplex micelles achieved significantly more efficient gene expression and cellular uptake compared with ligand-free PEG-micelles *in vitro* [26]. Micelles are normally formulated with biocompatible and biodegradable materials which makes them excellent nanocarrier systems [22]. The targeting ability of polymeric micelles is limited due to their low drug incorporation stability and low drug loading [27,28].

### 2.3. Dendrimers

Dendrimers differ from traditional polymers in the sense that they are highly branched synthetic polymers made of macromolecules such as poly (*N*-isopropylacrylamide)-polystyrene and poly(ethylene oxide)-poly(β-benzyl-L-aspartate) with an inner core diameter of less than 15 nm [9,29]. Dendrimers possess perfect nano-architecture composed of three different parts; a focal core, repetitive units of several interior layers, and multiple peripheral functional groups [30]. Because dendrimers are synthesized from branched monomers in a stepwise manner; it is possible to control some of their molecular properties including the shape, size, dimension, and, polarity [12]. Dendrimers offer enormous capacity for solubilization of hydrophobic compounds, and can be modified with guest molecules [31]. Therefore, dendrimers have shown enormous potential as anticancer drug delivery systems [32]. For example, Barker and coworkers produced dendrimers conjugated with fluorescein (FITC) and folic acid (FA) for imaging and therapeutic purposes [33]. In this study, dendrimers were linked with complementary DNA oligonucleotides to produce clustered molecules that target cancer cells overexpressing high-affinity folate receptors [33]. Limited number of preclinical or clinical studies of dendrimers as drug carriers is currently available. Thus, it is not possible to make any conclusions about the safety and/or efficacy of dendrimers for human use [34].

### 2.4. Quantum Dots

Quantum dots (QD) are small (2–10 nm) colloidal fluorescent semiconductor nanocrystals composed from 10–50 atoms of groups II–IV or III–V of the periodic table [29,35,36]. Their structure consists of a metalloid crystalline core and a shell that protect the core and renders the QD available for *in vivo* applications [37]. The size and shape of quantum dots can be controlled precisely, properties that determine their absorption and light emission [38]. One of the most valuable properties of QD is their fluorescence spectrum, which make them optimal fluorophores for biomedical imaging [37,39,40]. Fluorescent QD can be conjugated with bioactive moieties or specific ligands (e.g., receptor ligands and antibodies) [37]. QD are stable for months without degradation or alteration [12]. QD are mostly used as long-term, high-sensitivity and multicontrast imaging agents for detection and diagnosis of cancer *in vivo* [38]. Other examples of QD applications include transistors, solar cells, and quantum computing. Nevertheless, because they are composed of hazardous heavy metals, it is important to be cautious about their toxicity [37].

### 2.5. Fullerenes

Carbon nanotubes and buckyball clusters belong to the fullerenes, a family of structures composed entirely of carbon [29]. Carbon nanotubes are carbon coaxial graphite sheets of less than 100 nm rolled up into cylinders [29]. They can be classified in to two categories based on their structure: single-walled carbon nanotubes (SWNT) (one graphite sheet) or multi-walled carbon nanotubes (MWNT) (several concentric graphite sheets) [12]. They have been applied in biology as biosensors for detecting protein and DNA, diagnostics, and carriers [41]. This type of nanoparticle is insoluble in several solvents, provoking toxicity problems and some health concerns. However, they can be chemically modified to make them soluble in water, and functionalized so that they can be linked to active molecules such as nucleic acids, proteins, and therapeutic agents [41]. They have unique electronic, structural, and thermal characteristics that made them appropriate vehicles for drug delivery systems [12]. Liu *et al*. used single-walled carbon nanotubes (SWNT) chemically functionalized with PEG-paclitaxel (SWNT-PEG-PTX) in a xenograft breast cancer mouse model [42]. They observed higher tumor uptake of PTX and higher ratios of tumor to normal-organ PTX uptake for SWNT-PEG-PTX compared to taxol and PEG-PTX [42]. They also showed effective *in vivo* delivery of SWNT-PEG-PTX with higher tumor suppression efficacy and minimum side effects than taxol [42]. Due to their physicochemical properties, carbon nanotubes have additional applications in the computer, aerospace, electronics, and other industries [12,43]. Buckyball fullerenes have been tested *in vitro* as carriers for conventional anticancer agents (i.e. fullerene-paclitaxel conjugates) [44] and nucleic acids [45]. However there is striking evidence that fullerenes can cause oxidative damage to cellular membranes, and thus, toxicity [46,47]. The *in vivo* efficacy and safety of fullerenes require further studies.

### 2.6. Polymeric Nanofibers

Polymeric nanofibers describes fibers with diameters from 1 nm to 1 μm, closely matching the size scale of extracellular matrix (ECM) fibers [48,49]. Polymeric fibers are derived of inorganic (*i.e.*, titanium, silicon or aluminum oxides) or organic (polyvinyl alcohol, gelatin, poly(*N*-isopropylacrylamide, polycaprolactone, or polyurethane) materials. There are three available techniques for the synthesis of nanofibers: electrospinning, phase separation and self-assembly; however, the most commonly used is electrospinning [50]. Nanofibers consist of large surface area, low density, high pore volume, and tight pore size [51]. In addition, these properties can be easily changed by voltage, capillary collector distance, and polymer flow rate [52]. The surface tension and viscoelasticity of nanofibers in solution can also be modified [52]. Nanofibers are used for several applications such as medical (tissue engineering), filtration, barriers, wipes, personal care, composite, insulation, garments, and energy storage [51]. They have also been used as drug delivery systems. For example, Tseng and coworkers used biodegradable nanofibers to successfully deliver vancomicyn, an antibiotic, to the brain tissue of rats and reduce the toxicity associated with parenteral antibiotic treatment [53]. However, there are very few examples using polymeric nanofibers as cancer drug carriers.

### 2.7. Metal-Based Nanoparticles

Metal-based nanoparticles of different shapes, sizes (between 10 to 100 nm) have also been investigated as diagnostic and drug delivery systems. Most common metallic nanoparticles include gold, nickel, silver, iron oxide, zinc oxide, gadolinium, and titanium dioxide particles [54]. The large surface area of metallic nanoparticles enable the incorporation of high drug doses [55,56,57]. Qian *et al*. demonstrated the utility of gold-based nanoparticles in human cancer cells and in xenograft tumor mouse models. They reported the use of biocompatible and nontoxic PEG-gold nanoparticles for *in vivo* tumor targeting which were spectroscopically detected by surface-enhanced Raman scattering (SERS) [58]. Even though metallic nanoparticles are biocompatible and inert vehicles, a significant fraction of metal particles can be retained and accumulated in the body after drug administration, possibly causing toxicity [1]. Therefore, the use of metallic nanoparticles for drug delivery is a concern.

### 2.8. Nanoliposomes

Liposomes and particularly nanoliposomes are one of the most used delivery systems for small molecules, peptides, small and long nucleic acids, and proteins [59]. Liposomes were the first nanoparticle platform applied in medicine since Bangham described them in 1961 [6,60]. Nanoliposomes are nanometric (30–100 nm) versions of liposomes formed by expontaneous self-organization of phospholipids such as phosphatidylcholine, phosphatidylethanolamine, phosphatidylglycerol and phosphatidylserine, and other molecules such as cholesterol [9,59,61]. Importantly, many of the lipids used for liposome preparation are major components of naturally occurring bilayers [61]. The key common characteristic of bilayer-forming molecules is their defined polar and nonpolar regions that allows hydrophobic drugs to be embedded in the lipid bilayer, or be encapsulated in the central aqueous cavity when the molecule is hydrophilic [62]. Nanoliposomes have been used in medicine, biology, biochemistry, and in food and cosmetics industries [9,62,63].

Liposomes for therapeutic purposes are commonly manufactured using lipids, cholesterol and drug in a specific ratio. Functional groups are generally covalently bound to PEG which is normally 5% mol/mol of the total lipid [64]. To ensure a homogenous mixture of the liposome formulation, chloroform or chloroform:methanol are used as solvents. However, for therapeutic purposes, the use of chloroform is objectionable and tertiary butanol or cyclohexanes are used as alternatives. After freezing completely, the frozen lipid cake is placed on a vacuum pump and lyophilized until dry. Dry lipid films or cakes are then hydrated to obtain onion-like multilamellar vesicles (MLV). Small unilamelar vesicles (30–100 nm) are obtained by sonication or extrusion of MLV [65]. When injected in tumor-bearing mice, liposomes are distributed in the tumor, spleen and liver. However, liposomes are also detected in kidney, lung, and heart [66].

**Figure 1:**
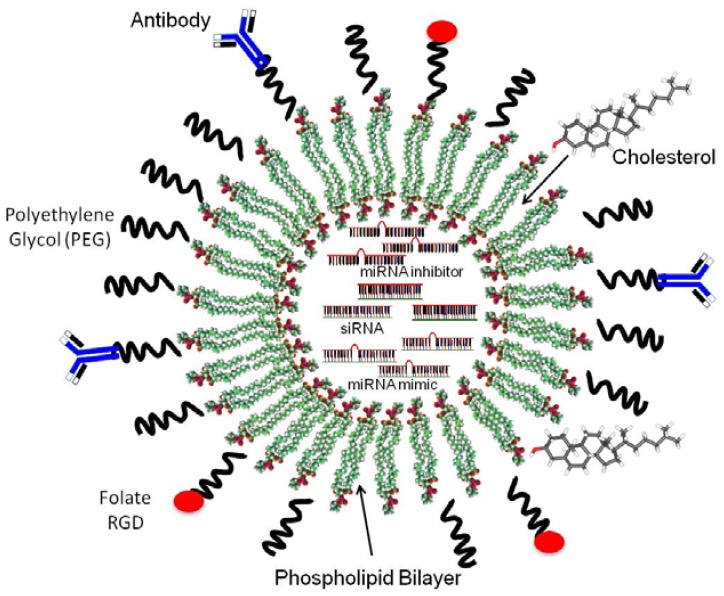
Nanoliposomes for drug delivery.

As drug carriers, nanoliposomes are able to increase the in vivo drug stability and bioavailability, by preventing interactions of the transported drug with unwanted molecules, and reducing toxic side effects [59]. Nanoliposomes offer the extra advantages of low toxicity, ability to modify size and surface, biocompatibility, and biodegradability [67]. In order to reduce the recognition of liposomes by macrophages, liposomes can be coated with biocompatible polymers like polyethylene glycol (PEG) (known as PEGylated or stealth liposomes). This strategy has greatly increased the liposomal stability and circulation half-time [7]. Furthermore, to increase accumulation in the desired target tissue, liposomes are surface attached with ligands capable of recognizing and binding to a particular group of cells (*i.e.*, the herceptin/trastuzumab antibody which targets the Her-2 antigen expressed by certain breast cancer cells, folate to target folate receptors which are overexpressed in a subset of ovarian cancer cells, or RGD to target integrins that are upregulated by proliferating endothelial cells of the tumor vasculature) [68,69] (Figure 1).

A liposome is a vesicle composed of a lipid bilayer. Liposomes are made of phospholipids and small amounts of other molecules as cholesterol and/or PEG (PEGylated or stealth liposomes). Functional groups (targeted liposomes) improve the specificity of the nanoparticle. Functional groups are generally covalently bound to PEG. SiRNA, miRNA inhibitors (antagomiRs) and miRNA mimics are encapsulated in the nanoliposome.

Currently, around fifteen liposomal-drug formulations for different conditions are in clinical use [9]. For cancer treatment, some examples include, DaunoXome (liposomal daunorubicin) for blood tumors, Doxil and Lipod-dox (PEGylated liposomal doxorubicin) for ovarian and breast cancers, and for Kaposi’s sarcoma patients [1]. Nab-paclitaxel (Abraxane) represents one of the new strategies to overcome the solvent-related problems of paclitaxel, and was recently approved by the US Food and Drug Administration (FDA) for pretreated metastatic breast cancer patients [70]. Additionally, several liposomal formulations are in different clinical trial phases. For, example, nanoliposomal CPT-11, a Phase I study, is used for patients with recurrent high-grade gliomas [71]. CPT-11 is a multi-component liposomal formulation containing a camptothecin derivate and a topoisomerase-I inhibitor [72]. Other liposomal drug formulations include, SPI-077 (liposomal cisplatin for solid tumors), CPX-351 (cytarabine: daunorubicin for acute myeloid leukemia), Lipoplatin (cisplatin for non-small cell lung cancer), ThermoDox (a thermosensitive doxorubicin for hepatocellular carcinoma, and other advanced cancers), and Stimulax (an anti-MUC1 cancer vaccine for non-small cell lung cancer). In addition, Yakult Honsha Co., Ltd. developed IHL-305, a PEGylated liposome containing irinotecan [73]. IHL-305 is currently in a phase I study for advanced solid tumors [74].

## 3. The Small Interference RNA (siRNA) Strategy in Cancer Medicine

Although small molecules currently used as chemotherapeutic agents have led to numerous successful therapies for cancer; they are not able to discriminate between cancerous and non-cancerous cells. Thus, the development of alternative approaches for specific targets in cancer cells is highly desirable. Furthermore, many intracellular pathways become deregulated in cancer cells, and the use of two or more chemotherapeutic agents targeting more than one deregulated pathways is a rational therapeutic strategy. Therefore, the use of RNA interference (RNAi) to downregulate multiple targets has emerged as a treatment modality of exceptional promise for cancer treatment [75]. Basically, the RNAi strategy uses small RNA molecules which bind to messenger RNAs (mRNA) by complementary base pairing, to induce degradation of the mRNA and/or block protein synthesis. The small RNA molecules are incorporated and processed into cellular RNA processing machinery to induce their inhibitory effects. In one RNAi-based therapy modality, a 21-27 base pair double stranded RNA (siRNAs) is introduced into the cells, where it binds to its specific complementary mRNA sequence and inhibits protein synthesis (this effect is commonly called RNA silencing) [76]. SiRNAs are designed to target only one gene which is generally overexpressed in cancer cells compared with normal cells [76]. Therefore, it is highly desirable to use siRNAs targeting key genes involved in cancer cell migration, invasion, and metastasis.

A more recent related modality is to introduce 22-bp single or double stranded RNAs into the cells to manipulate the microRNAs (miRNAs). MiRNAs are endogenously expressed small non-coding RNAs (21- to 25-nucleotides in length) that function as post-transcriptional regulators of gene expression [77,78]. Recent evidence indicates that the human genome may encode over 1500 miRNAs which regulate about 40%–60% of the human genes [79]. Multiple studies involving various types of human cancers have demonstrated that miRNAs have a fundamental role in tumorigenesis and drug resistance [80,81,82,83,84,85,86]. A particular miRNA can potentially bind to thousands of mRNAs. While some miRNAs are down-regulated and act as tumor suppressor genes, others are up-regulated and may represent novel oncogenes (oncomiRs) [8]. Thus, miRNA-targeted therapies are used in two ways, to inhibit the action of miRNAs via oncomiRs (antagomirs) or to imitate the function of tumor suppressor genes (microRNA mimics). One of the most attractive properties of miRNAs as therapeutic agents, and probably the most important advantage in comparison with siRNA approaches, is that while siRNA-based approaches target single genes, the miRNA-based approaches target multiple molecules, making them highly efficient in regulating distinct biological processes relevant to normal and malignant cells [87]. However, in some tissues certain miRNAs could regulate a group of genes, each one with opposite roles [88].

Before RNAi discovery, sequence-specific inhibition of gene expression was achievable using nucleic acid-based antisense technology [89]. One of the major advantages of RNAi over antisense based approaches is that the RNAi relies on the cellular machinery to target complementary transcripts resulting in efficient and potent down-regulation of gene expression [89]. Furthermore, the use of the RNAi strategy is highly specific when modulation of a particular target is desired. In this scenario, lower side effects are expected compared to conventional chemotherapy. In spite of all the advantages of RNAi as a therapeutic strategy in cancer medicine, the *in vivo* systemic administration of RNAi has remained a major challenge due to its short half-life [90], activation of the immune response, lack of ability to penetrate the plasma membrane, and potential toxicity [91]. Therefore, the use of nanoparticles as RNAi carriers has been proposed to address these concerns.

### 3.1. Pre-Clinical Studies Using RNAi-Loaded Nanoliposomes

Several RNAi-loaded cationic, anionic or neutral nanoliposomes have been tested in animal models to demonstrate the potential of the RNAi therapeutic modality [89,92]. However, very few of them have advanced to clinical trials. For example, Landen Jr. *et al*. have developed a 1,2-dioleoyl-sn-glycero-3-phosphocholine (DOPC)-based neutral nanoliposome formulation with siRNA targeted to EphA2, a receptor highly abundant in ovarian cancer [93]. Multiple doses of this formulation reduced tumor growth in mouse models of ovarian cancer, and is currently in clinical trials (see below) [93]. Saltzman and co-workers developed and used a PLGA nanoparticle composed of FDA-approved materials with mitogen-activated protein kinase (MAPK1)-targeted siRNA in the mouse female reproductive tract [94]. A single dose of MAPK1-targeted siRNA-loaded nanoparticles caused efficient and sustained gene silencing [94]. Saltzman and co-workers demonstrated the effectiveness of biodegradable polymer nanoparticles as drug delivery vehicles for siRNA in the mouse vaginal mucosa [94]. Chen *et al*. designed a nanoparticle with 1,2-distearoyl-*sn*-glycero-3-phosphoethanolamine-*N*-[folate(polyethylene glycol)-5000] (DSPE-PEG-5000) and an asparagine-glycine-arginine peptide to target aminopeptidase N (CD13), a protein involved in cancer invasion, angiogenesis, and metastasis. This nanoparticle also carried c-MYC-targeted siRNA and doxorubicin [95]. Three injections of this formulation resulted in a partial inhibition of tumor growth in tumor-bearing mice [95]. In addition, Tanaka et al used a multistage system composed of mesoporous silicon particles loaded with DOPC-based neutral nanoliposomes, containing siRNA against the EphA2 oncoprotein, which is overexpressed in several cancers, including ovarian and breast cancers [96]. A single injection of this formulation achieved a significant reduction of tumor growth in ovarian cancer mouse models [96]. This study represent the first *in vivo* therapeutic validation of a novel, multistage siRNA delivery system for sustained gene silencing with broad applicability to pathologies beyond ovarian neoplasms [96]. Using a similar approach, Vivas-Mejía *et al.* used a siRNA targeting the splice variant 2B of survivin, an antiapoptotic protein highly abundant in several cancers [97]. Multiple injections of this DOPC-based nanoliposome formulation reduced tumor growth in xenograft mouse models of taxane-resistant ovarian cancer [97]. This research team proposed the use of survivin 2B-siRNA-DOPC as a novel and specific anti-survivin therapy without compromising all intracellular pools of survivin in normal tissues [97]. Several other siRNA-loaded nanoliposome formulations have been tested in animal models of virtually all cancer types, including prostate [98], breast [99], ovarian [93], pancreatic [98], and melanoma [99].

Although nanoliposomes are the most versatile carriers for systemic siRNA delivery, other nanoparticle formulations are also being investigated, including chitosan, polyethyleneimine, PLGA, dendrimers, and metal-based nanoparticles [99,100,101]. Particularly, Han *et al*. used RGD-labeled chitosan nanoparticles containing a siRNA against plexin domain-containing protein 1 (PLXDC1), a protein upregulated in the ovarian cancer vasculature [102]. This approach resulted in significant inhibition of tumor growth of ovarian cancer mouse models [103].

Significant advances have been made in the development of miRNA-based therapies for cancer and other human diseases. For example, Mirna Therapeutics™ has initiated a Phase 1 clinical study with a miR-34 mimic (see below). In addition, this company is performing pre-clinical studies with a Let-7 mimic for non-small cell lung cancer (NSCLC). [104]. *In vivo* experiments of exogenous delivery of let-7 blocked lung tumor growth by repressing proliferation and eliminating tumor cells through a non-apoptotic mechanism [105,106]. Similarly, the systemic delivery of miR-34 mimics blocked tumor growth in mouse models with NSCLC and prostate cancer [107,108]. In addition, Asuragen is working on the first miRNA-based diagnostic test for distinguishing pancreatic cancer from chronic pancreatitis. Several miRNAs have also been developed in other areas such as cardiovascular diseases and hepatitis C (HCV) [104]. For example, miR-195, has been linked to pathological cardiac growth and its overexpression resulted in heart failure in transgenic mice [109]. Likewise, miR-208, a cardiac-specific miRNA, is required for cardiomyocyte hypertrophy, fibrosis, and expression of β*MHC*, a primary contractile protein of the heart [110]. Santaris Pharma A/S sponsored phase 2 clinical studies with a miR-122 inhibitor (Miravirsen), and reported prolonged reduction of the hepatitis-C virus after 4-weeks patient treatment.

Other nanoparticle formulations for systemic delivery of miRNA mimics and antagomiRs are also being investigated [105,106,107,108,109,111]. For example, Liang *et al.* developed PLGA-based nanoparticles polyplexed with a miRNA expression vector [112]. This new approach for gene therapy by miRNA delivery via PLGA/PEI nanoparticles resulted in enhanced cellular uptake, and pronounced up-regulation of the miRNA, induction of cell cycle arrest, and improved gene delivery in human hepatocellular carcinoma cells [112]. Furthermore, Piao *et al.* developed a lipid-based nanoparticle for the delivery of pre-miR-107 that inhibits the tumorigenicity of head and neck squamous cell carcinoma [113].

### 3.2. Clinical Trials

Six siRNA-containing nanoparticles for cancer therapy entered to Phase I clinical trial (Table 1) [99]. The first siRNA phase I trial CALAA-01 was developed by Calando Pharmaceuticals against solid tumors [99,114].

**Table 1:** SiRNA/miRNA Cancer therapeutics in clinical trials.

Drug	Target	Disease	Company	Stage
SiRNA Cancer therapeutics in clinical trials				
CALAA-01	M2 subunit of ribonucleotide	Solid tumors	Calando Pharmaceuticals	Ongoing Phase I, Not recruiting
ALN-VSP02	VEGF and KSP	Solid tumors involving the liver	Alnylam Pharmaceuticals	Completed Phase I
Atu027	Protein Kinase 3 (PKN3)	Solid tumors	Silence Therapeutics AG	Completed Phase I
TKM 080301	Polo-like kinase 1	Solid tumors	Tekmira Pharmaceutical	Recruiting Phase I
siG12D LODER	KRAS	Pancreatic ductal adenocarcinoma	Silenseed Ltd	Phase II, Not yet open
siRNA-EphA2-DOPC	EPHA2	Solid tumors	M.D. Anderson Cancer Center	Phase I, not yet open
**MiRNA Cancer therapeutics in clinical trials**				
MRX34	miR-34 mimic	Liver cancer or metastatic cancer with liver involvement	Mirna Therapeutic, Inc.	Recruiting Phase I

CALAA-01 is a siRNA (targeting M2 subunit of the ribonucleotide reductase complex) in an amantadine-cyclodextrin vehicle that contains PEG-shielding and employs transferrin (Tf) as a targeting ligand [115]. After the development of CALLA-01, several other companies, including Alnylam, Tekmira, Silence Therapeutics, Marina and others, introduced siRNA nanoparticles products into preclinical and clinical phases [99]. For example, Alnylam Pharmaceuticals has the ALN-VSP02, a siRNA-carrying liposomal formulation for liver-related solid tumors [116]. ALN-VSP02 contains two siRNAs targets against vascular endothelia growth factor (VEGF) and kinesin spindle protein (KSP). This siRNA-liposomal formulation already completed the phase I stage. Silence Therapeutics AG completed the phase I stage for its liposomal siRNA formulation, Atu027, used to treat advanced solid tumors including gastrointestinal and lung cancers [117]. The active ingredient of Atu027 is a siRNA against protein kinase 3 (PKN3), a kinase involved in metastatic motility [98]. Tekmira Pharmaceutical Corporation has a phase I trial for dose-escalation of TKM 080301, a lipid nanoparticle formulation with a siRNA against polo-like kinase 1 for patients with solid tumors [118]. Silenseed Ltd has initiated a Phase II trial to evaluate the progression-free survival (PFS) of patients treated with siG12D LODER (Local Drug EluteR) [119]. SIG12D LODER is a polymeric-based matrix with a siRNA against the mutated KRAS oncogene, which is mutated and overexpressed in more than 90% of human pancreatic ductal adenocarcinomas. Mirna Therapeutics, Inc., a biopharmaceutical company focused on microRNA-directed oncology therapies, recently initiated the first Phase 1 clinical trial of MRX34 [120]. MiRX34 is a liposome-formulated miRNA mimic of the tumor suppressor miR-34, for use in patients with liver cancer or metastatic cancer with liver involvement. *In vivo* evidence showed that miR-34 mimic induces cell cycle arrest, senescence, and cell death, and affects the viability of cancer stem cells, creates a barrier to metastasis, and sensitizes cancer cells to other therapies [121]. This study is currently recruiting participants to evaluate MRX34 safety. Finally, the M.D. Anderson Cancer Center is sponsoring a phase I clinical trial to test the safety and highest tolerable dose of siRNA-EPHA2-DOPC for patients with advanced, recurrent ovarian cancer [122].

## 4. Conclusions and Future Directions

The development of nanoparticle formulations as drug delivery systems has greatly improved the stability and therapeutic effectiveness of several anticancer agents. As drug carriers, nanoliposomes are preferred over other nanoparticle platforms because of their biocompatibility, biodegradability, low cost, stability, long circulating times (PEGylated liposomes), and high encapsulation efficiency. In addition, nanoliposomes can entrap both lipophilic and hydrophilic molecules, and they can be functionalized by attaching specific targeting ligands to their external surface. The development of RNAi technology holds great potential as a therapeutic strategy in cancer medicine. However, various concerns must be overcome to definitely demonstrate the safely and efficacy of the nanoparticle-RNAi modality in the clinical setting:

(1) Identification of more precise molecules/pathways required for tumor development and growth [123,124]. For example, the design of siRNAs targeting genes highly abundant in cancerous cells or in certain cancer stem cells populations is desirable [97,124];
(2) As most of the studies assessing the biological and molecular effects of targeting miRNAs have been performed in cells in culture, and in some animal models, more *in vivo* studies of the therapeutic consequences of miRNA-based therapies are required. Likewise, as miRNAs are regulated in a tissue-specific and stage-specific manner [75,76,77,78,79,80], the choice of the correct miRNA as a target, is another aspect of paramount importance in the design of effective miRNA-based nanoliposomal formulations for cance
(3) Identification of specific receptors in the surface of cancer cells for the creation of targeted nanoparticle-RNAi delivery systems (double targeting) [125]. Targeting specific cell surface receptors is achievable by the direct conjugation of anticancer drugs with specific ligands or with the use of ligand-PEG-derivatized lipids in nanoliposomes [11,95];
(4) Additional pharmacokinetic, pharmacodynamics, and tissue distribution studies of the nanoparticle and nanoparticle-RNAi formulations [126]. Results of these studies will demonstrate the safety of the liposomal-RNAi formulations for cancer patients. The development of most sensitive methods to calculate the amounts of siRNA, miRNA inhibitors, and miRNA mimics in blood, urine, tumors, and other organs is also needed [127,128];
(5) Elaboration of easy administration (oral and spray) of nanoparticle-RNAi formulations. These delivery methods may be advantageous in terms of cost and for patient’s quality of life [129,130];
(6) Dose adjustment studies, especially when double targeting is desirable. These studies should guarantee that appropriate concentrations of RNAi, ligands, and lipids will be delivered into the tumor tissue, and other organs [131]. Moreover, therapies capable of crossing the blood-brain barrier are also required for treatment of brain cancers. The nanoparticle-RNAi modality is slowly moving into the clinic not only for cancer but for many other conditions. Results of the ongoing clinical trials will confirm whether the nanoliposomal RNAi strategy are safe and effective for cancer treatment. It is anticipated that in the near future the nanoparticle-RNAi modality will bring more and improved therapeutic options for several human diseases.
